# Development and evaluation of a customized checklist to assess the quality control of disease registry systems of Tehran, the capital of Iran in 2021

**DOI:** 10.1186/s12913-023-09605-2

**Published:** 2023-07-04

**Authors:** Maryam Barzin, Hamideh Sabbaghi, Sharareh Kamfar, Atena Seifi, Mahmoud Hajipour, Fatemeh Hadavand Siri, Elham Mir-Moeini, Anis Gharajeh, Nasrin Ferdosifard, Mohammadhossein Panahi, Seyed Saeed Hashemi Nazari, Fatemeh Fallah Atatalab, Koorosh Etemad

**Affiliations:** 1 Obesity Research Center, Research Institute for Endocrine Sciences, Shahid Beheshti University of Medical Sciences, Tehran, Iran; 2 Ophthalmic Epidemiology Research Center, Research Institute for Ophthalmology and Vision Science, Shahid Beheshti University of Medical Sciences, Tehran, Iran; 3 Department of Optometry, School of Rehabilitation, Shahid Beheshti University of Medical Sciences, Tehran, Iran; 4 Pediatric Congenital Hematologic Disorders Research Center, Research Institute for Children’s Health, Shahid Beheshti University of Medical Sciences, Tehran, Iran; 5 Pediatric Nephrology Research Center, Research Institute for Children’s Health, Shahid Beheshti University of Medical Sciences, Tehran, Iran; 6 School of Public Health and Safety, Shahid Beheshti University of Medical Sciences, Tehran, Iran; 7 Cancer Research Center, Shahid Beheshti University of Medical Sciences, Tehran, Iran; 8 Tracheal Diseases Research Center, National Research Institute of Tuberculosis and Lung Diseases, Masih Daneshvari Hospital, Shahid Beheshti University of Medical Sciences, Tehran, Iran; 9 Department of Epidemiology, School of Public Health and Safety, Shahid Beheshti University of Medical Sciences, Tehran, Iran; 10 Department of Epidemiology, School of Public Health and Safety, Epidemiology, Safety Promotion and Injury Prevention Research Center, Shahid Beheshti University of Medical Sciences, Tehran, Iran; 11 Safety Promotion and Injury Prevention Research Center, Shahid Beheshti University of Medical Sciences, Tehran, Iran

**Keywords:** Checklist, Quality control, Disease registry system, Iran

## Abstract

**Background:**

Clinical registries facilitate medical research by providing ‘real data’. In the past decade, an increasing number of disease registry systems (DRS) have been initiated in Iran. Here, we assessed the quality control (QC) of the data recorded in the DRS established by Shahid Beheshti University of Medical Sciences in Tehran, the capital city of Iran, in 2021.

**Methods:**

The present study was conducted in two consecutive qualitative and quantitative phases and employed a mixed-method design. A checklist containing 23 questions was developed based on a consensus reached following several panel group discussions, whose face content and construct validities were confirmed. Cronbach’s alpha was calculated to verify the tool’s internal consistency. Overall, the QC of 49 DRS was assessed in six dimensions, including completeness, timeliness, accessibility, validity, comparability, and interpretability. The seventy percent of the mean score was considered a cut-point for desirable domains.

**Results:**

The total content validity index (CVI) was obtained as 0.79, which is a reasonable level. Cronbach’s alpha coefficients obtained showed acceptable internal consistency for all of the six QC domains. The data recorded in the registries included different aspects of diagnosis/treatment (81.6%) and treatment quality requirements outcomes (12.2%). According to the acceptable quality cut-point, out of 49 evaluated registries, 48(98%), 46(94%), 41(84%), and 38(77.5%), fulfilled desirable quality scores in terms of interpretability, accessibility, completeness, and comparability, however, 36(73.5%) and 32(65.3%) of registries obtained the quality requirement for timeliness and validity, respectively.

**Conclusion:**

The checklist developed here, containing customized questions to assess six QC domains of DRSs, provided a valid and reliable tool that could be considered as a proof-of-concept for future investigations. The clinical data available in the studied DRSs fulfilled desirable levels in terms of interpretability, accessibility, comparability, and completeness; however, timeliness and validity of these registries needed to be improved.

## Introduction

Clinical registries are interactive real-time databases recording the detailed information of patients, including specific diagnoses, clinical conditions, and procedures [1]. A clinical registry is typically customized to fulfill its major purposes, including describing the natural history of diseases, treatments, medications, and their clinical efficacy and cost-effectiveness, as well as disease outcomes and safety and quality of care issues [2, 3]. In 1974, the World Health Organization (WHO) pioneered these registries in epidemiological and clinical research [4], and since then disease registry systems (DRSs) have turned into organized systems to improve the quality of care in the healthcare system and progress in medical research [5, 6]. Disease registry systems provide opportunities to conduct high-quality medical research, advance diagnostic and therapeutic clinical practice guidelines, and improve the quality of healthcare services, patient outcomes, and resource and financial management. In addition, DRSs can show us the best way to strategic purchasing and conduct cohort and clinical trials based on real data [7, 8], especially for rare diseases [9].

However, using databases for research and audit and answering specific questions require high-quality data and resolving the weaknesses of DRSs [10]. Quality control (QC), as an integrated system, is an important component of the quality management of data registries, helping in dynamic monitoring and evaluating the effectiveness of the construct and process of data registration according to predetermined goals. As with any project, DRSs should be evaluated by a supervisor appointed by the founder. This process helps DRSs improve themselves and correct their errors, such as missing data, delayed follow-up, and data duplication [4, 11, 12]. Therefore, clinical DRSs need to be validated and improved as their quality assessment results are provided to policymakers and health insurance companies and are used for making public-heath related decisions.

In Iran, DRSs started to grow 30 years ago when the cancer registry and then other national registries such as trauma, spinal defects, and newborn anomalies were established [13–16]. In 2014, a national DRS program was created aiming to integrate at least 20 DRSs in Iran. Until November 2018, a total of 71 clinical DRSs were active in the medical universities and health institutions affiliated with Iran’s Ministry of Health and Medical Education, among which Shahid Beheshti University of Medical Sciences (SBMU), with six DRSs, ranked fifth until then. This university now hosts around 50 DRSs for different medical specialties. The pillar of dynamic monitoring of these DRSs is to validate their different QC dimensions and integrate them into a meaningful whole to provide comprehensive coverage while keeping these dimensions organized and integrating them with the health information system (HIS).

Because it is inapplicable to interpolate all internationally used QC dimensions into all DRSs, we here developed a checklist to monitor the most important QC dimensions including comparability [17], reliability, and validity [18], completeness [19], timeliness [11], accessibility [20], efficiency, and duplication [2]. The second purpose was to evaluate the quality of 49 DRSs.

## Methods

The present study had a mixed-method design and was conducted in two consecutive qualitative and quantitative phases. In the qualitative phase, a checklist was developed to assess the QC of 49 active DRSs established by the research centers, hospitals, and educational departments affiliated with the Shahid Beheshti University of Medical Sciences (SBMU). In these DRSs, the data were launched using unique and standard software, and crude data were transferred from actively supervised registries approved by SBMU. Registries with no recorded data were excluded.

### Ethical consideration

This study’s procedures were approved by the Ethics Committee of SBMU under the registration number IR.SBMU.RETECH.REC.1400.577.

### Qualitative phase: checklist development

A working group consisting of 13 experts was formed to develop a checklist through a panel group discussion and brainstorming. The working group’s members included epidemiologists (*n* = 3), medical informatics specialists (*n* = 3), social medicine specialists (*n* = 3), health policymakers with experience in the field of DRSs (*n* = 2), and two DRS professionals. First, an initial form of the checklist was designed based on the standards released by the Ministry of Health of Iran, and then it was further updated based on the key points extracted from relevant articles retrieved by systematically searching different databases. Finally, a checklist with 29 items was developed, and each item was scored on a five-point Likert scale from "not important" (one score) to "very important" (five scores). A total of three panel-group discussion sessions were held with the participation of all 13 experts. During the first session, questions with scores of 4 or 5 were kept and further examined in the subsequent panel-group sessions, and questions obtaining a score of < 4 were omitted. At the end of the third panel-group discussion, a checklist with 23 items was finalized.

The questions were developed in a way to evaluate different QC aspects of the registries, including structure management, data sources, data elements, registry software, recording processes, registry outcomes, user training, and the performance of the QC subcommittee.

The final checklist approved by the panel group was presented to an examiner team, whose members had no previous encounter with the research topic, to assess the face validity and understandability of the questions. In this step, content validity was numerically calculated using two indicators: Content Validity Index (CVI) and Content Validity Ratio (CVR). Items with CVI scores less than 0.7 were omitted. Considering that our expert team had 13 members, the acceptable CVR was designated above 0.56 based on the Lawshe “minimum CVR value”. The reliability of the checklist was verified based on Cronbach’s α.

### Training courses

In this step, eight examiners were requested to designate the level of QC for each item of the checklist based on an organized guideline presented to them during two training courses. The examiner team consisted of individuals who were registrars, researchers, executive directors, quality experts, administrators, and supervisors who were familiar with the process of data registration. All examiners (*n* = 8) participated in the training courses with a total duration of four hours. Finally, the QC of each registry was separately checked by the examiners using the provided checklist. During the training courses, the executive director of the registry provided the related documents and reports and briefly explained data collection methods. All examiners were then asked to rate each QC dimension for the registry according to the checklist under the supervision of the head of the team. All examiners independently investigated the registry. In order to calculate the agreement between examiners, the checklist was completed twice for two of the registries (# 25 and #26) at an interval of three months between June and August 2021. The average of Kappa agreement obtained was beyond 80%, which is considered acceptable.

### Quantitative phase: data collection

During the evaluation step, four examiner teams assessed the QC of the registries from August to November 2021. On the examination day, the executive director presented the annual reports of the registry to examiner teams. Then the registry was rated for different items available on the checklist.

### Data quality dimensions

There is a need to accurately define and regularly monitor all QC dimensions, some of which have been well discussed and defined in various fields of medicine [17]. Comparability, completeness, and validity are considered key QC items in most registries [21]. In the present study, we focused on six QC areas to assess our DRSs, including 1) *Comparability*: intra-organizational consistency of data over time allowing for comparison [17]; 2) *Completeness*: the data collected matching the data expected to describe a specific entity [19]; 3) *Timeliness*: collecting and sharing data within a reasonable time to be used for intended purposes [11]; 4) *Interpretability*: ease of understanding the data [22]; 5) *Accessibility*: ease of access to the data; users’ being informed of what data are being collected and where they are located; and 6) *Validity*: adhering to the rules or definitions applicable to the data during data collection [2].

A rating scale of 0 to 650 was used to rank the data during quality analysis. This rating scale was then transformed into a percentage system from 0 (the lowest quality) to 100% (the highest quality). A cut-point of 70% of the mean score based on a consensus reached by the panel group was considered desirable for different QC domains, including completeness, timeliness, accessibility, validity, comparability, and interpretability, whose individual cut-points were obtained as 111.3, 45.8, 70.4, 26.3, 5.6, and 20.57, respectively.

## Results

During the study’s qualitative phase, a total of 29 items were extracted corresponding to the goals of the Ministry of Health and according to the literature in order to rank the quality of each registry. A satisfactory level of agreement was observed among the panelists with regard to the final 23 QC items. Regarding content validity assessment, the CVI and CVR of these 23 items were 0.79 and 0.58, respectively, suggesting the good content validity of the checklist items. Cronbach’s alpha coefficients for all QC domains were higher than 0.69, indicating acceptable internal consistency. This checklist was used to evaluate six QC domains for DRSs, including completeness (Q1, Q2, Q3, Q5, Q6, Q7, Q10, Q17, and Q18), timeliness (Q11, Q21, and Q22), accessibility (Q12, Q20, and Q23), validity (Q9, Q13, and Q14), comparability (Q16), and interpretability (Q4). These dimensions were ranked based on the total scores from the summing of related questions. Furthermore, data duplication, confidentiality, and understanding of the memorandum (MOU) were separately evaluated by the questions of Q8, Q15, and Q19, respectively (Table 1).

**Table 1:**
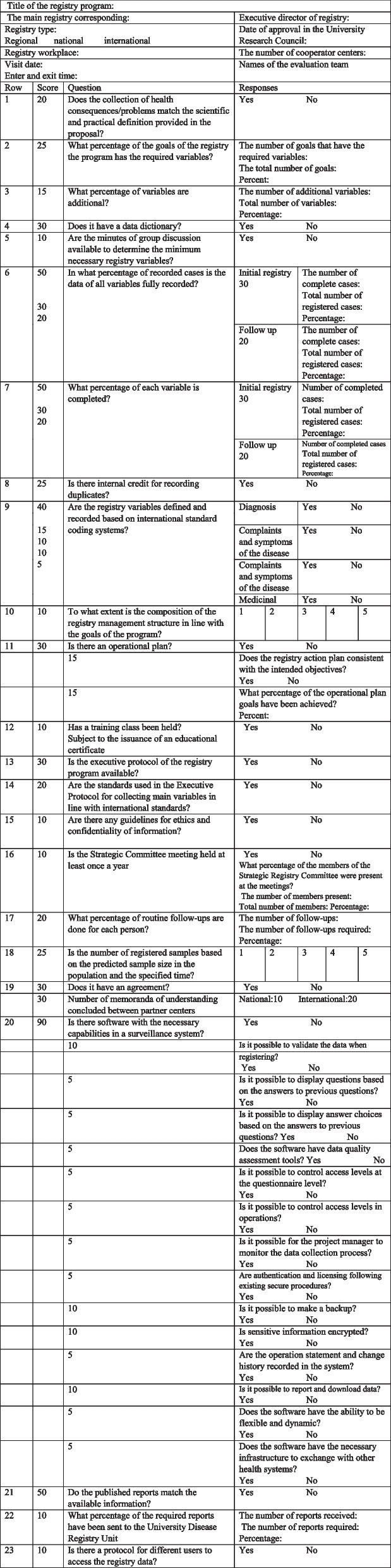
Quality control checklist for disease registry programs

**Fig. 1:**
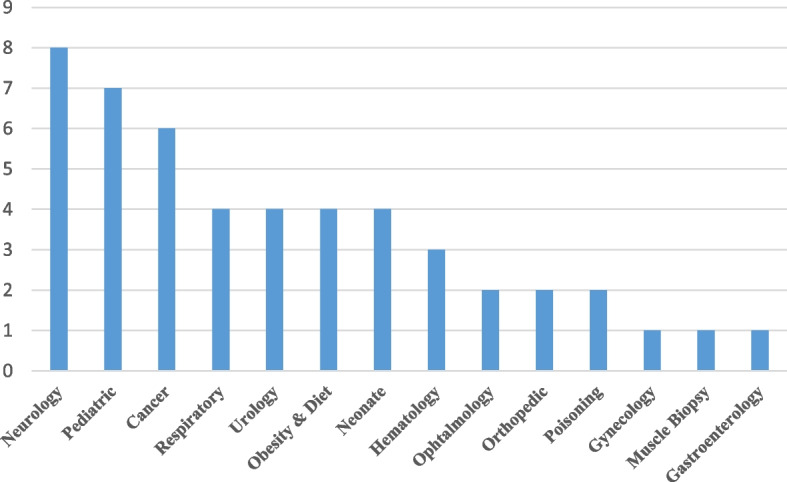
Registries established by Shahid Beheshti University of Medical Sciences, Iran, in different health fields

During the study’s quantitative phase, the registries were assigned specific serial numbers. An overview of the 49 registries approved by SBMU has been shown in Table 2. Most of the registries (81.6%, *n* = 40) were focused on diagnosis/treatment; six of them (12.2%) recorded treatment outcomes and other registries were related to different scopes of diagnosis (*n* = 1,#49), procedures (*n* = 1, #47), and side effects of treatments (*n* = 1, #30). As illustrated in Fig. 1, most of the registries were in the field of neurology (*n* = 8), followed by pediatrics (*n* = 7) and cancer management (*n* = 6).

**Table 2:** Overview of 49 quality control registry’s in Shahid Beheshti University of medical science

**Serial No**	**Registry name**	**Registry field**	**Registry subfield**	**Participation in healthcare units**	**Year of establishment**	**Level**	**Coverage area**	**Last cumulative report of registered cases**
**Neurology**								
#1	Parkinson's Disease Registry in Patients Referred to Neurology Clinics of SBMU-PDR	Neurology	Diagnosis-Treatment	Outpatient	2019	National	• Tehran, SBMU • Ilam • Semnan • Zabol • Isfahan • Ardabil • Izeh	1000
#2	Iranian Registry of Patients with Spinal Muscular Atrophy (SMA)	Physical Medicine and Rehabilitation	Diagnosis-Treatment	Outpatient	2020	Regional	• Tehran, SBMU • Tehran, IMU	19
#3	Brachial Plexus Damages Patients, Records In 15thkhordad Hospital /Tehran /Iran	Plastic & Reconstructive Surgery	Diagnosis-Treatment	Inpatient	2019	Regional	• Tehran, SBMU	116
#4	Multiple Sclerosis	Neurology	Diagnosis-Treatment	Outpatient	2014	Regional	• Tehran, SBMU • Karj, AMU	-
#5	Iranian Registry of Neuro-metabolic Disease	Pediatric Neurology	Diagnosis-Treatment	Outpatient	2012	National	• Tehran, SBMU • Tehran, TMU • Tabriz, TMU • North Khorasan University of Medical Sciences • Isfahan,	250
#6	Registry of patients with Neurocutaneous syndromes	Pediatric Neurology	Diagnosis-Treatment	Inpatient	2020	Regional	• Tehran, SBMU	55
#7	Registry of Refractory Epilepsy in Iranian Children	Pediatric Neurology	Diagnosis-Treatment	Outpatient	2019	Regional	Shahid Beheshti University of Medical Sciences	180
#8	The National Pediatric Migraine Registry	Pediatric Neurology	Diagnosis-Treatment	Outpatient	2020	Regional	• Tehran, SBMU • Tehran, TMU	130
**Serial No**	**Registry name**	**Registry field**	**Registry subfield**	**Participation in healthcare units**	**Year of establishment**	**Level**	**Coverage area**	**Last cumulative report of registered cases**
**Cancer**								
#9	Cancer registry Using GIS system	Pulmonary Diseases	Treatment	Outpatient	2010	Regional	• Tehran, SBMU	-
#10	Breast cancer clinical registry in Iran	Surgical Oncology	Diagnosis-Treatment	Outpatient	2000	Regional	• Tehran, SBMU	1000
#11	Thyroid nodule and differentiated thyroid cancer registry	Endocrinology & Metabolism	Diagnosis-Treatment	Inpatient	2020	Regional	• Tehran, SBMU	96
#12	Monitoring of intra-oral potentially malignant disorders	Oral & Maxillofacial Medicine	Diagnosis-Treatment	Outpatient	2020	National	• Tehran, SBMU • Tehran, BMU • Birjand, • Babol • Zahedan	130
#13	Colorectal Cancer Registry System	Gastroenterology and Hepatology	Diagnosis-Treatment	Inpatient	2020	Regional	• Tehran, SBMU	450
#14	Pancreatic Cancer Registry System	Gastroenterology and Hepatology	Diagnosis-Treatment	Inpatient	2020	Regional	• Tehran, SBMU	250
**Respiratory**								
#15	National registry program of Post Intubation Tracheal Intubation	Thoracic Surgery	Treatment	Inpatient	211	National	• Tehran, SBMU	3000
#16	Chronic Obstructive Pulmonary Diseases Registry in Masih Daneshvari Hospital	Pulmonary Diseases	Treatment	Inpatient	2017	Regional	• Tehran, SBMU	-
#17	Iranian-based registry for pulmonary arterial hypertension: Using GIS system	Pulmonary Diseases	Treatment	Outpatient	2010	Regional	• Tehran, SBMU	846
#18	Bronchopulmonary dysplasia patients registration system in hospitals	Neonatal-Perinatal Medicine	Diagnosis-Treatment	Inpatient	2020	Regional	• Tehran, SBMU	100
**Serial No**	**Registry name**	**Registry field**	**Registry subfield**	**Participation in healthcare units**	**Year of establishment**	**Level**	**Coverage area**	**Last cumulative report of registered cases**
**Urology**								
#19	Registry of patients with urinary system stones	Urology	Diagnosis-Treatment	Outpatient	2020	Regional	• Tehran, SBMU	4638
#20	Recurrent Urinary Tract Infection in Children	Pediatric Nephrology	Diagnosis-Treatment	Outpatient	2020	Regional	• Tehran, SBMU	83
#21	Pediatric nephrotic syndrome children	Pediatric Nephrology	Diagnosis-Treatment	Inpatient	2020	Regional	• Tehran, SBMU	80
#22	A national registry system for patients undergone Reconstructive Urologic procedures	Urology	Diagnosis-Treatment	Inpatient	1997	National	• Tehran, SBMU • Urmia • Ahvaz • Tabriz • Mashhad	2700
**Orthopedic**								
#23	Database registry for hip arthroplasty	Hip & Pelvis Surgery	Diagnosis-Treatment	Inpatient	2020	Regional	• Tehran, SBMU	80
#24	Database registry for knee arthroplasty	Orthopedics	Diagnosis-Treatment	Inpatient	2020	Regional	• Tehran, SBMU	79
**Ophthalmology**								
#25	The National Registry for Keratoconus in Iran	Cornea & External Eye Diseases	Diagnosis-Treatment	Outpatient	2019	Regional	• Tehran, SBMU	600
#26	The First Database Registry for Hereditary Retinal Dystrophies and Degenerations in Iran	Retina & Vitreous	Treatment	Outpatient	2016	National	• Tehran, SBMU • Tehran, TMU • Tehran, IMU • Ahvaz • Tabriz • Mashhad • Isfahan • Yazd	2100
**Serial No**	**Registry name**	**Registry field**	**Registry subfield**	**Participation in healthcare units**	**Year of establishment**	**Level**	**Coverage area**	**Last cumulative report of registered cases**
**Neonatal**								
#27	Kernicterus registry system in the hospitals	Neonatal-Perinatal Medicine	Diagnosis-Treatment	Inpatient/ Outpatient	2021	Regional	• Tehran, SBMU	-
#28	Neonatal thrombosis registration system	Neonatal-Perinatal Medicine	Diagnosis-Treatment	Inpatient	2020	Regional	• Tehran, SBMU	26
#29	Very Low Birth Weight Infants Registration system	Neonatal-Perinatal Medicine	Diagnosis-Treatment	Inpatient	2020	Regional	• Tehran, SBMU	200
#30	Register the BCG vaccine complications in pediatric	Vaccination	Side effects	Outpatient (PHC)	2019	Regional	• Tehran, SBMU	16
**Pediatric**								
#31	Iranian Registry of Pediatric Inflammatory Bowel Disease (PIBD)	Pediatrics Gastroenterology	Diagnosis-Treatment	Outpatient	2019	National	• Tehran, SBMU • Babol • Hamedan • Urmia • Isfahan • Ilam	61
#32	Registry of Pediatric Wilson Disease	Pediatrics Gastroenterology	Diagnosis-Treatment	Outpatient	2020	National	• Tehran, SBMU • Babol • Hamedan • Urmia • Isfahan • Ilam • Ghom	107
#33	Registry of pediatric autoimmune hepatitis	Pediatrics Gastroenterology	Diagnosis-Treatment	Outpatient	2019	National	• Tehran, SBMU • Babol • Hamedan • Urmia • Isfahan • Ilam • Ghom	56
**Serial No**	**Registry name**	**Registry field**	**Registry subfield**	**Participation in healthcare units**	**Year of establishment**	**Level**	**Coverage area**	**Last cumulative report of registered cases**
#34	Iranian Registry of pediatric endoscopy databases system clinical outcomes research initiative procedures and liver biopsy	Pediatrics Gastroenterology	Diagnosis-Treatment	Inpatient	2019	National	• Tehran, SBMU • Babol • Hamedan • Urmia • Isfahan • Ilam	702
#35	Registry system of Autistic patients	Pediatrics Neurology	Diagnosis-Treatment	Outpatient	2021	Regional	• Tehran, SBMU	50
#36	Pediatric Liver Failure (pALF) Registration System in Iran	Pediatrics Gastroenterology	Diagnosis-Treatment	Outpatient	2019	National	• Tehran, SBMU • Babol • Hamedan • Urmia • Isfahan • Ilam	28
#37	Registry system for evaluation of childhood fatty liver in Iran	Nutritional Sciences	Diagnosis-Treatment	Inpatient	2019	Regional	• Tehran, SBMU	19
**Obesity & Diet**								
#38	Registry of ketogenic diets	Pediatric Neurology	Treatment	Outpatient	2020	National	• Tehran, SBMU • Tehran, TMU • Tabriz • Isfahan	87
#39	Registry system for evaluation of childhood Obesity in Iran	Pediatrics Gastroenterology	Diagnosis-Treatment	Outpatient	2019	National	• Tehran, SBMU • Babol • Hamedan • Urmia • Isfahan • Ilam • Ghom	158
#40	Registry system for evaluation of the malnutritional status of children and adolescents hospitalized in Iran (1 month to 18 years)	Nutritional Sciences	Diagnosis-Treatment	Inpatient	2019	National	• Tehran, SBMU • Babol • Hamedan • Urmia • Isfahan • Ilam • Ghom	63
**Serial No**	**Registry name**	**Registry field**	**Registry subfield**	**Participation in healthcare units**	**Year of establishment**	**Level**	**Coverage area**	**Last cumulative report of registered cases**
#41	Registration of patients in Tehran Obesity Treatment Center	Obesity	Diagnosis-Treatment	Inpatient	2014	Regional	• Tehran, SBMU	4600
**Hematology**								
#42	Data registry of Hematopoietic Stem Cell Transplantation in pediatrics group (0–18 years old)	Pediatric Hematology and Oncology	Diagnosis-Treatment	Inpatient	2020	Regional	• Tehran, SBMU	129
#43	Regional Registry of Pediatric Immune Thrombocytopenic Purpura	Pediatric Hematology and Oncology	Diagnosis-Treatment	Inpatient	2020	Regional	• Tehran, SBMU	10
#44	Registry of thromboembolism events in pediatrics group (up to15 years)	Pediatric Hematology and Oncology	Diagnosis-Treatment	Inpatient	2020	Regional	Shahid Beheshti University of Medical Sciences	25
**Poisoning**								
#45	Poisoning information registry system	Pediatrics Poisoning	Diagnosis-Treatment	Inpatient	2021	Regional	• Tehran, SBMU	90
#46	Iranian Registry of Pediatric Lead Poisoning	Pediatrics Gastroenterology	Diagnosis-Treatment	Inpatient	2019	National	• Tehran, SBMU • Babol • Hamedan • Urmia • Isfahan • Ilam	97
**Gastroenterology**								
#47	Establishment of Registry System for ERCP (Endoscopic Retrograde Cholangio Pancreatography)	Gastroenterology and Hepatology	Procedure	Inpatient	2020	Regional	• Tehran, SBMU	1400
**Gynecology**								
#48	Premature ovarian failure	Gynecology	Diagnosis-Treatment	Outpatient	2020	Regional	• Tehran, SBMU	200
**Muscle Biopsy**								
#49	Registry of Patients Referred for Muscle Biopsy	Muscle Biopsy	Diagnosis	Outpatient	2020	Regional	• Tehran, SBMU	5000

Table 3 shows the ranking of the registries studied based on their QC scores. The highest rank (96.1%) belonged to registries #22 and #42, and the lowest ranking (20%) was related to registry #49.

**Table 3:** Total score and rank obtained of disease registry programs of Shahid Beheshti University of Medical Sciences in 2021

Serial No	Registry program title	Total score	Score of 100	Ranking
#42	Data registry of Hematopoietic Stem Cell Transplantation in pediatrics group (0–18 years old)	625	96.1	1
#22	A national registry system for patients undergoing reconstructive urologic procedures	625	96.1	1
#43	Regional Registry of Pediatric Immune Thrombocytopenic Purpura	615	94.6	2
#44	Registry of thromboembolism events in pediatrics group (up to15 years)	605	93.1	3
#40	Registry system for evaluation of the malnutritional status of children and adolescents hospitalized in Iran (1 month to 18 years)	605	93.1	3
#36	Pediatric Liver Failure (pALF) Registration System in Iran	605	93.1	3
#41	Registration of patients in Tehran Obesity Treatment Center	605	93.1	3
#2	Iranian Registry of Patients with Spinal Muscular Atrophy (SMA)	605	93.1	3
#10	Breast cancer clinical registry in Iran	600	92.3	4
#39	Registry system for evaluation of childhood Obesity in Iran	590	90.1	5
#20	Recurrent Urinary Tract Infection in Children	585	90.0	6
#12	Monitoring of intra-oral potentially malignant disorders	585	90.0	6
#15	National Registry program of Post Intubation Tracheal Intubation (ALBORZ database)	580	89.2	7
#25	The National Registry for Keratoconus in Iran	575	88.4	8
#21	Pediatric nephrotic syndrome children	570	87.7	9
#18	Bronchopulmonary dysplasia patients registration system in hospitals	565	86.9	10
#8	The National Pediatric Migraine Registry	565	86.9	10
#26	The First Database Registry for Hereditary Retinal Dystrophies and Degenerations in Iran	555	85.4	11
#29	Very Low Birth Weight Infants Registration system	550	84.6	12
#38	Registry of ketogenic diets	545	83.8	13
#23	Database registry for hip arthroplasty	540	83.1	14
#33	Registry of pediatric autoimmune hepatitis	505	77.7	15
#37	Registry system for evaluation of childhood fatty liver in Iran	495	76.1	16
#28	Neonatal thrombosis registration system	490	75.3	17
#32	Registry of Pediatric Wilson Disease	480	73.8	18
#34	Iranian Registry of pediatric endoscopy databases system clinical outcomes research initiative procedures and liver biopsy	480	73.8	18
#17	Iranian-based registry for pulmonary arterial hypertension: Using GIS system	475	73.1	19
#46	Iranian Registry of Pediatric Lead Poisoning	470	72.3	20
#1	Parkinson's Disease Registry in Patients Referred to Neurology Clinics of SBMU-PDR	470	72.3	20
#35	Registry system of Autistic patients	440	67.7	21
#6	Registry of patients with Neurocutaneous syndromes	435	66.9	22
#3	Brachial Plexus Damages Patients, Records In 15thkhordad Hospital /Tehran /Iran	420	64.6	23
#16	Chronic Obstructive Pulmonary Diseases Registry	410	63.1	24
#24	Database registry for knee arthroplasty	400	61.5	25
#30	Register the BCG vaccine complications in pediatric	390	60.0	26
#7	Registry of Refractory Epilepsy in Iranian Children	375	57.7	27
#45	Poisoning information registry system	370	56.9	28
#31	Iranian Registry of Pediatric Inflammatory Bowel Disease (PIBD)	370	56.9	28
#14	Pancreatic Cancer Registry System	350	53.8	29
#27	Kernicterus registry system in the hospitals	340	52.3	30
#5	Iranian Registry of Neuro-metabolic Disease	315	48.5	31
#47	Establishment of Registry System for ERCP (Endoscopic Retrograde Cholangio Pancreatography)	310	47.7	32
#13	Colorectal Cancer Registry System	310	47.7	32
#4	Multiple Sclerosis	305	46.9	33
#48	Premature ovarian failure	285	43.8	34
#11	Thyroid nodule and differentiated thyroid cancer registry	265	40.7	35
#9	Cancer registry Using GIS system	260	40.0	36
#19	Registry of patients with urinary system stones	190	29.2	37
#49	Registry of Patients Referred for Muscle Biopsy	130	20.0	38

Table 4 presents the mean score of each QC domain for DRSs based on the total scores from the summing of related questions. Regarding the acceptable quality cut-point (i.e., > 70% of the mean score of each domain), out of 49 DRSs evaluated, 48 (98%), 46 (94%), 41 (84%), 38 (77.5%), 36 (73.5%), and 32 (65.3%) registries obtained quality scores in the domains of interpretability, accessibility, completeness, comparability, timeliness, and validity, respectively. In this study, the rate of recording duplicated data was low (12.2%), which can be explained by the development of electronic registries with unique national ID numbers.

**Table 4:** Scores of six domains of quality control of 49 registries

Serial No	**Registry Title**	**Comparability**	**Completeness**	**Timeliness**	**Accessibility**	**Interpretability**	**validity**
	Mean score of each domain of 49 registries ± SD	7.9 ± 3.7	159.0 ± 56.0	65.4 ± 29.5	100.5 ± 25.0	29.4 ± 4.3	37.6 ± 27.1
	70% of the mean score as cut-point	5.6	111.3	45.8	70.4	20.6	26.3
#1	Parkinson's Disease Registry in Patients Referred to Neurology Clinics of Shahid Beheshti University of Medical Sciences in Tehran (SBMU-PDR)	10	135	85	100	30	15
#2	Iranian Registry of Patients with Spinal Muscular Atrophy (SMA)	5	210	90	110	30	65
#3	Brachial Plexus Damages Patients, Records In 15thkhordad Hospital /Tehran /Iran	10	145	90	110	30	0
#4	Multiple Sclerosis	10	130	0	100	30	0
#5	Iranian Registry of neurometabolic patients	10	110	35	10	30	50
#6	Registry of patients with Neurocutaneous syndromes	10	180	20	110	30	50
#7	Registry of Refractory Epilepsy in Iranian Children	10	130	30	110	30	30
#8	The National Pediatric Migraine Registry	10	205	85	110	30	50
#9	Establishment of Cancer Registry in Masih Daneshvari Hospital: Using GIS system	0	50	55	100	30	15
#10	Establishment of Breast cancer clinical registry in Iran	10	225	85	110	30	45
#11	Thyroid nodule and differentiated thyroid cancer registry	0	55	5	100	30	0
#12	Setting up of a registry system and monitoring of intra-oral potentially malignant disorders in Shahid Beheshti Dental School	10	210	80	110	30	50
#13	Establishment of colorectal cancer Registry System	0	95	60	100	30	0
#14	Establishment of Pancreatic Cancer Registry System	0	125	60	100	30	0
#15	National registry program of Post Intubation Tracheal Stenosis (ALBORZ database)	5	225	85	110	30	90
#16	Chronic Obstructive Pulmonary Diseases Registry in Masih Daneshvari Hospital	10	180	30	100	30	50
#17	Iranian-based registry for pulmonary arterial hypertension: Using GIS system	10	185	85	100	30	30
#18	Establishment of bronchopulmonary dysplasia patients registration system in hospitals of Shahid Beheshti University of Medical Sciences	10	200	85	110	30	45
Serial No	**Registry Title**	**Comparability**	**Completeness**	**Timeliness**	**Accessibility**	**Interpretability**	**validity**
#19	Registry of patients with urinary system stones	10	55	0	75	0	15
#20	Recurrent Urinary Tract Infection in Children	5	205	85	110	30	65
#21	Pediatric nephrotic syndrome	10	205	90	110	30	30
#22	Establishment of a national registry system for patients undergone Reconstructive Urologic procedures	10	225	90	110	30	65
#23	Establishment of the database registry for hip arthroplasty	10	205	85	110	30	65
#24	Establishment of the database registry for knee arthroplasty	10	115	85	110	30	15
#25	The National Registry for Keratoconus in Iran	10	180	85	110	30	65
#26	Establishment of the First Database Registry for Hereditary Retinal Dystrophies and Degenerations in Iran	10	145	75	110	30	90
#27	Establishment of a kernicterus registry system in the hospitals affiliated with Shahid Beheshti University of Medical Sciences	10	80	30	100	30	15
#28	Establishment of the neonatal thrombosis registration system in Shahid Beheshti University of Medical Sciences	10	125	75	110	30	45
#29	Establishment of Very Low Birth Weight Infants Registration system	10	225	75	110	30	15
#30	Register the BCG vaccine complications in pediatric	10	145	60	110	30	65
#31	Iranian Registry of Pediatric Inflammatory Bowel Disease (PIBD)	10	95	10	110	30	45
#32	Registry of Pediatric Wilson Disease	10	145	90	110	30	0
#33	Registry of pediatric autoimmune hepatitis	10	180	25	110	30	50
#34	Iranian Registry of pediatric endoscopy databases system clinical outcomes research initiative procedures and liver biopsy	10	120	85	110	30	30
#35	Registry system of Autistic patients	10	180	25	110	30	50
#36	Establishment of Pediatric Liver Failure (pALF) Registration System in Iran	10	205	90	110	30	65
#37	Registry system for evaluation of childhood fatty liver in Iran	10	205	90	110	30	65
#38	Registry of ketogenic diets	5	190	90	110	30	50
#39	Registry system for evaluation of childhood Obesity in Iran	10	195	85	110	30	65
#40	Registry system for evaluation of the malnutritional status of children and adolescents hospitalized in Iran (1 month to 18 years)	10	210	85	110	30	65
#41	Registration of patients in Tehran Obesity Treatment Center	10	225	90	110	30	65
Serial No	**Registry Title**	**Comparability**	**Completeness**	**Timeliness**	**Accessibility**	**Interpretability**	**validity**
#42	Data registry of Hematopoietic Stem Cell Transplantation in pediatrics group (0–18 years old) in Shahid Beheshti University of Medical Sciences and Allied centers	10	225	90	110	30	65
#43	Regional Registry of Pediatric Immune Thrombocytopenic Purpura	10	215	90	110	30	65
#44	Data registry of thromboembolism events in pediatrics group (up to15 years) in Mofid Children`s Hospital and allied centers hospital in Iran	10	125	75	110	30	45
#45	Designing and Implementation of poisoning information registry system	0	150	55	100	30	0
#46	Iranian Registry of Pediatric Lead Poisoning	5	140	90	110	30	0
#47	Establishment of Registry System for ERCP (Endoscopic Retrograde CholangioPancreatography)	0	120	10	100	30	15
#48	premature ovarian failure	10	180	30	10	30	15
#49	Registry of Patients Referred for Muscle Biopsy	0	25	50	0	30	0

## Discussion

In this study, we developed a customized checklist to assess six quality domains in 49 DRSs established by SBMU in 2021. Our results demonstrated that all the registries had acceptable interpretability, accessibility, comparability, and completeness. In addition, the timeliness and validity domains acquired the lowest quality ranks.

There are several methodological problems with data quality assessment, one of which is the lack of a comprehensive standardized method for this purpose. The quality of data varies between different practices, and data quality needs to be assessed based on unique requirements in various fields [21, 23, 24]. Comparability, completeness, and validity are considered key elements during data quality assessment [21, 23]. Faulconer and de Lusignan suggested an 8-step statistical method for assessing the quality of the data of patients with chronic obstructive pulmonary disease [24]. Therefore, we developed a specific checklist to assess the QC of our DRSs in different fields.

Only a high degree of completeness will ensure that the incidence and prevalence rates estimated in DRSs are close to their ‘true’ values. Most data QC dimensions overlap with each other, and their interpretations are vague due to ambiguous definitions or even a lack of standard definitions. Two of the most frequently cited data QC dimensions are “accuracy” and “completeness”. It may be difficult to accurately estimate the completeness of registries because this entity is influenced by the proportion of patients introduced to registries by healthcare centers, the ratio of those refusing to be referred, and the total number of patients in the study population [25]. In our investigation, the overall rate of completeness was obtained at 84%, which was in line with the study conducted by Fung et al*.* on Singapore’s cancer registries, reporting a completeness rate of 98.1% [26]. One possible reason for lower completeness in some of our DRSs may be the short time passing from their establishment (#11, #19, and #27). Despite all the limitations such as the relatively short period of the study, in a study by Lee et al*.*, completeness of 90–100% was reported for a registry of operative sectors (e.g., operating surgeons, consulting surgeons, and the hospital). Interestingly, auditing revealed that the registry’s completeness reached 100% after resolving deficiencies [27]. Another explanation for this variation in completeness may be differences in the number of patients with specific disorders such as Parkinson's disease (#1), SMA (#2), pediatric migraine (#8), tracheal stenosis following intubation (#15), and pediatric nephrotic syndrome (#21). In addition, nationwide recruitment for a number of our registries (#42, #43, and #44) could have contributed to their high completeness rates. Also, some medical procedures should be registered before their costs can be reimbursed by insurance companies (registries #12, #23, and #24). Another factor increasing the completeness of data recording can be the proven utility of this practice amongst health professionals in the registeration centers (#10, #18, #22, #36, #37, and #41). It is worth noting that using this checklist, we were unable to determine the proportion of eligible patients who decided not to be enlisted in relevant registries, increasing the likelihood of overestimating completeness in these registries.

Using standard internationally approved definitions for recording and reporting data boosts the level of comparability of registries [11]. In our study, there was limited standardization regarding the definitions used in registries, leading 11 out of 49 registries to have unacceptable comparability in terms of diagnostic and therapeutic elements. It is worth mentioning that in our checklist, only one question (Q16) was related to comparability, limiting the ability of this checklist to reliably assess this quality domain compared to other dimensions investigated by multiple questions. Comparability has been reported to vary considerably in different registries. In a study in Russia, only four cancer registries out of 10 studied registries met international standards [28]. However, cancer registries in Singapore were reported to have a high level of comparability [26]. Low comparability is the main barrier to achieving an interoperability framework, and one potential solution to this problem is to develop a team of specialists and experts to standardize definitions across all DRSs.

High timeliness allows for the real-time recording of diagnoses, procedures, and other relevant data in DRSs. Although there are currently no international regulations for assessing timeliness, timely reporting of information is a foremost priority for all registries [1, 11]. There are rare reports on the timeliness of DRSs [29], and the definition of this term in the context of data registries should be exactly determined. In general, timeliness refers to the rapidity at which a registry can collect, process, and report reliable and complete data [30]. In our checklist, timeliness was defined as the date on which the database was ‘frozen’ to calculate annual statistics for issuing an official report. This period comprised two intervals: the time until receipt announcement (ie., from the date of diagnosis to the day of receiving the report) and the processing time (i.e., from the date of report receiving until data availability). In our report, in 36 out of 49 registries (73.5%), physicians or nurses reported the data at the time of patient visits or shortly afterward, but five registries had delays in submitting annual reports. Also, eight of the registries preferred to postpone the publication of their results to attain better completeness to the cost of undesirable timeliness. Evaluating the timeliness and several other quality dimensions of a pediatric mortality surveillance system in Iran showed that this system successfully fulfilled timeliness criteria mainly because the managers were committed to holding a monthly committee for monitoring childhood mortality and the immediate reporting of infectious diseases [31]. Moreover, an evaluation of timeliness at the Cancer Registry of Norway during 1953–2005 showed that the median time for diagnosis of a new case reduced from over 525 days in 2001 to 261 days in 2005 [29]. Another study showed that the timeliness of the diseases was low based on the national reporting of the disease surveillance system [32]. Therefore, implementing electronic data recording and employing dedicated and well-trained staff can improve the timeliness of registries in reporting their data.

Accessibility is defined as the ease of access to data for users, rendering the data more available and making it possible for others to confirm the registry’s results [33]. As almost all of our registries used the same unique software, they acquired high accessibility (94%). This unique software facilitates the generation of meaningful and credible information from diverse sources, decreasing the occurrence of potential errors during the data entry process and facilitating access to the data. In line with our results, a study by Azadmanjir et al*.* aimed to identify and address hurdles to data accessibility at the National Spinal Cord Injury Registry of Iran, a registry relying on primary data sources. Their expert panel selected 174 data quality items, including accessibility and usefulness in quality-of-care assessment in emergency settings [34].

Validity is the extent to which the data registered can be assessed in terms of accuracy and relevant rules or definitions [35]. A high validity rate (91.9%) was reported for Singapore’s cancer registries, which could be attributed to the fact that these registries were focused on a specific field [26]. In the present study, nearly one-third of our registries (35%) had low validity, mainly due to the lack of uniform definitions for items due to the variety of DRS fields. This observation highlights the importance of employing uniform, transparent, and accurate definitions by all registries according to existing guidelines and classifications.

Interpretability is defined as the ease of understanding of data for users, presenting one of the main challenges faced by data registries and requiring a clinical framework for data interpretation and scoring [2]. Registered data can be interpreted only if they are specifically assigned to exact endpoints [22]. In our checklist, this quality domain was assessed by a single question (Q4), asking about the presence or absence of a data dictionary at each registry, and almost all (98%) of the registries investigated obtained a score higher than the cut-point. There are no previous reports on the interpretability of registered data in DRSs, so we could not compare our observation with other studies in terms of this QC domain.

The main strength of the present investigation included the development of a customized checklist to assess data quality in Iran’s DRSs in various fields in terms of all main QC standard indicators. One of the limitations of this study was that we confined our search to DRSs established by SBMU in Tehran, which could decrease the generalizability of our findings. Second, most of these DRSs mainly rely on the reports generated by governmental hospitals, and it is possible that the private hospitals or health service centers in different regions might not be covered.

## Conclusion

In this study, a customized checklist was developed to assess the quality of the data recorded in DRSs, which could be considered a proof of concept for future investigations. Our results demonstrated that most of DRSs had high degrees of interpretability, accessibility, comparability, and completeness. However, their timeliness and validity needed to be improved. As the DRSs of the SBMU acquired a high degree of quality control in most of the studied domains allows for greater confidence in the use of the qualified data to improve the healthcare system and the possibility of integrating data with national healthcare data.

## Data Availability

The datasets analysed during the current study available from the corresponding author on reasonable request.
